# Needs, status and future directions in palliative care for children with cancer: an insight into the Italian situation

**DOI:** 10.3389/fonc.2025.1582341

**Published:** 2025-04-16

**Authors:** Cosimo Chelazzi, Daniele Marelli, Paola Mantovani, Francesca Franchi, Cesare Vezzoli

**Affiliations:** ^1^ Dipartimento di Specialità Medico-Chirurgiche, Scienze Radiologiche e Sanità Pubblica, Università degli Studi di Brescia, Brescia, Italy; ^2^ S.C. Cure Palliative e ADI/S.S. Cure Palliative Pediatriche, ASST Spedali Civili Brescia, Brescia, Italy

**Keywords:** pediatric palliative care, pediatric oncology, early integration, symptom management, healthcare models

## Abstract

Pediatric palliative care (PPC) is an essential component of holistic care for children with cancer, addressing the physical, emotional, psychosocial and existential needs of children and their families. This narrative review explores the status, challenges, and future directions of PPC for children with cancer, specifically focusing on the Italian context. Despite advancements in legislation, such as Italy’s Law 38/2010, access to PPC remains uneven, particularly in underserved regions. Children with cancer experience a high symptom burden, including pain, fatigue, and dyspnea, which is often inadequately managed due to late referrals and misconceptions equating PPC with end-of-life care. Organizational barriers, such as limited trained personnel and inadequate service models are linked to delayed integration. Physician discomfort, family resistance, and uncertainty regarding referral timing also contribute to delayed referral. International studies highlight regional disparities. However, opportunities for improvement exist, including workforce development, targeted education, and public awareness campaigns. Models of care, such as home-based services, telemedicine, and floating clinics, demonstrate potential for addressing gaps and optimizing resource allocation. Data from Italian centers further emphasize the importance of early PPC integration to improve outcomes, reduce unnecessary intensive care interventions, and ensure a better quality of life. This review underscores the need for tailored, multidisciplinary PPC models, proactive screening tools, and sustained policy support to ensure equitable and comprehensive care for all children with cancer.

## Introduction

The World Health Organization (WHO) defines pediatric palliative care (PPC) as an holistic approach to care that begins at the diagnosis of a life-threatening illness and continues irrespective of whether the child is receiving disease-directed treatments (1). PPC seeks to address physical, emotional, social, and spiritual needs, with a strong emphasis on family-centered care and recognition of the developmental differences between children and adults. Unlike adult palliative care, PPC often spans several years, adapting to the evolving needs of patients and families throughout the course of illness (2).

Children with cancer frequently experience severe symptoms, including pain, fatigue, and dyspnea, which are often inadequately managed (3, 4). These symptoms, along with others such as poor appetite and gastrointestinal issues, can be under-recognized by physicians, leading to communication gaps with parents. Early discussions about timely integration of palliative interventions are relevant, for improving quality of life and underscore the need for proactive PPC implementation.

The Italian Law 38/2010 is recognized internationally as a comprehensive legislative framework for palliative care (5). It guarantees access to palliative care and pain management as a fundamental right, including for children. The law mandates the development of regional PPC networks that must include specialized centers, pediatric hospices, and home-based care teams.

This narrative review aims to provide an example of the status, challenges, and future directions of PPC for children with cancer, with a specific focus on its integration into pediatric oncology care and the Italian situation.

## Methods

The papers for this narrative review were identified through a series of PubMed searches conducted between December 2024 and January 2025, using various combinations of relevant keywords, including “palliative care,” “pediatric palliative care,” “pediatric oncology,” “pediatric hemato-oncology,” “health models,” and “transition.” Only full-text articles published in English were considered. The final selection of papers was made through a collaborative discussion among all authors, based on their expertise and the relevance of the studies to the topic.

## Epidemiology and special needs

According to the World Health Organization (WHO), approximately 21 million children worldwide require pediatric palliative care (PPC) each year. Of these, more than 8 million need specialized PPC services (6). In high-income countries, pediatric cancer remains a notable cause of morbidity and mortality. Globally, over 4 million children with oncological conditions are estimated to require palliative care (7, 8), while up to 90% of childhood cancer cases occur in low- and middle-income countries (9).

In Italy, approximately 10,600 children require specialized PPC annually; around 1800 of these are estimated to be children with cancer (10).

In a retrospective analysis of 870 children referred from 2008 to 2022 to the specialist PPC referral center at the Padua Pediatric Hospital, children with cancer accounted for 24% of PPC referrals, comprising brain tumors (40%), solid tumors (38%), and liquid tumors (18%) (11). Children with oncological conditions showed a significantly higher mortality rate (90% vs. 41%) and a shorter follow-up duration, with 91% followed for less than 12 months (median: 1.8 months, 95% CI: 1.4–2.7) compared to 52% of non-oncological cases followed for over 12 months (median: 43 months, 95% CI: 37–47). Oncological patients also had a lower discharge rate (5% vs. 18%), reflecting the complexity and progression of their disease. The findings highlight the need for earlier palliative care integration for oncological patients and sustained care pathways for non-oncological cases (11). Data from other centers may be different even on a regional basis. In a survey conducted in the Italian region of Emilia-Romagna, the total number of children with life-limiting conditions was 601, with a mean age of 7.4 ± 4.8 years and a prevalence of 8.4 per 10,000, lower than the national estimates, and children with cancer accounted only for 4% of the total cohort (12). The reason for this may include different models of oncology-PPC integration and uneven availability of PPC services.

Moreover, the landscape is changing, towards a progressive better survival rate (11), with more than 80% of children with cancer achieving 5-year survival in high-income settings (7). However, a substantial proportion of survivors—up to 60%—will experience long-term health challenges, including organ dysfunction, secondary malignancies, and infertility, underscoring the potential role of ongoing palliative care support (7).

Adolescents and young adults (AYA) with cancer represent a special population, facing unique and often unmet palliative and end-of-life care needs. They endeavor high levels of distress, severe refractory symptoms, and frequent exposure to intensive medical measures at the end of life (13). Tailored psychosocial support is critical, requiring care adapted to their independence and maturity while fostering peer relationships and minimizing isolation through physical or digital spaces (14). Age-appropriate, multidisciplinary care can address many unmet needs, but families also require regular interdisciplinary support, particularly regarding anticipatory grief.

## Needs, outcomes and barriers for pediatric palliative care in oncology

Prevalence and intensity of symptoms burden is consistent across different countries.

The pioneering study by Wolfe et al. provided a comprehensive analysis of the symptom burden and challenges in PPC for children with cancer, particularly at the end of life (3). Interviews with 103 parents of children who died of cancer (1990–1997) revealed that 89% experienced severe symptoms, including pain, fatigue, and dyspnea, with symptom management often inadequate (successful control in only 27% of pain and 16% of dyspnea cases). Parents reported more symptoms, such as fatigue, poor appetite, and gastrointestinal issues, than were documented by physicians, highlighting under-recognition and communication gaps. Pain was more prevalent when physicians were less involved in end-of-life care (OR 2.6; 95% CI: 1.0–6.7). Early discussions about hospice and timely integration of palliative interventions were associated with improved quality of life, stressing the need for proactive PPC implementation (3).

The same findings were confirmed by Olagunju et al., who conducted a cross-sectional study of 72 child-caregiver pairs in a resource-limited setting (9). Children with advanced cancer experience a significant symptom burden, including lack of energy (68%), pain (62%), nausea (56%), worry (51%), and difficulty sleeping (49%). Other prevalent symptoms include lack of appetite (48%), weight loss (36%), hair loss (34%), and feelings of sadness (32%). The global symptom burden and individual child symptoms in children were correlated positively with depressive symptoms in caregivers (P<0.05), 38.2% of whom screened positive for depression (9).

The outcome of PPC in alleviating symptoms and improving quality of life of children with cancer is well-documented, as demonstrated by Kaye et al. in their systematic review of 32 studies involving 15,635 pediatric oncology patients (15). PPC involvement significantly enhanced symptom management and facilitated advance care planning, while also reducing intensive care use and increasing the likelihood of dying at home, in accordance with family preferences. Moreover, PPC improved communication between families and healthcare teams, leading to greater satisfaction with the overall care provided.

Children with cancer experience a significant symptom burden, even in the early stages of treatment, underscoring the need for early palliative care (PC) integration to: address physical, emotional, and psychosocial challenges; enhance outcomes and quality of life for patients and families (16). Patients generally view early PC positively, with little resistance or negative impact on hope, therapy, or the relationship with their oncologists (16).

Indeed, caring for the family is quintessential to PPC respect to adults PC, with a special focus on caregiver training and respite care (2). Addressing the unique social and educational needs of pediatric patients through community integration, such as school coordination is another central scope for PPC teams.

The transition from pediatric to adult palliative care is an additional, peculiar challenge of PPC. This transition requires interdisciplinary collaboration and careful planning to ensure continuity of care. Barriers to this process include the loss of familiar care teams and inadequate communication between pediatric and adult providers. Structured transition programs and individualized care plans have been recommended to address these challenges and support young adults in navigating the complexities of new healthcare systems (17).

Despite this evidence on outcomes, access to PPC remains inconsistent and the late integration of PPC into cancer care often restricts its role to end-of-life care (11, 18).

Cheng et al. (19) conducted a systematic review of 16 studies, encompassing 3,796 pediatric oncology patients, to evaluate the timing of palliative care initiation. The review found that PC discussions occurred a mean of 509.6 days after cancer diagnosis, and PC initiation typically took place close to death, with a mean duration of care of 57.8 days before death. Only 54.5% of patients received any PC services before death, highlighting significant delays in integration (19).

The early referral to PPC may result in better advance care planning along the trajectory of the cancer, reducing the risk of futile medical treatments, including admissions to hospitals and intensive care units (ICUs) at the end-of-life.

Kaye et al. conducted a retrospective cohort study of 321 pediatric oncology patients enrolled in PPC at St. Jude Children’s Research Hospital between 2011 and 2015 (20). Results showed that 79.4% of patients received experimental therapies, and 35.5% continued cancer-directed treatments in the last month of life. Moreover, 51,4% of the children were ICUs, and 44.3% of in-hospital deaths occurred in ICUs. Children enrolled into PPC less than 30 days before death were nearly five times more likely to die in ICUs. Taylor et al. conducted a mixed-methods systematic review of 42 studies to evaluate the impact of specialist PPC on children with cancer and identify barriers to access (21). Results showed that PPC involvement was associated with a 22% reduction in intensive care unit admissions and a 30% increase in advance care planning. Additionally, PPC involvement reduced in-hospital deaths by 18%, aligning care with family preferences.

Bereavement support is part of the care that should be provided to the families and the surviving siblings (8). However, a structured approach to this is often lacking, and PPC are in the ideal position to provide this support, provided their involvement has been timely. Wiener et al. surveyed 100 healthcare professionals, primarily palliative care physicians (51%) and oncologists (19%), to assess bereavement care practices in pediatric oncology (22). The study found that 59% of centers often or always provide bereavement care, but only 5% use formal assessment tools, and 28% lack systematic follow-up with families. Common practices included sending condolence cards (80%) and making phone calls (72%). Barriers to care included insufficient policies and staff resources, highlighting the need for standardized guidelines to support grieving families effectively.

## Challenges and opportunities

The integration of PPC faces numerous challenges but also presents key opportunities for improvement.

A qualitative study with four focus groups of 31 pediatric oncology providers to examine barriers and facilitators to early PPC integration (23). Issues included role overlap, conflicting philosophies between PPC and curative/oncologic care, concerns about patient readiness, and emotional challenges for physicians. The key facilitators identified were clear eligibility criteria, improved patient care, education on PPC, and evidence-based approaches. Physicians were less supportive of early PPC, while nurses and social workers emphasized its necessity for unmet symptoms and psychosocial needs.

A recent survey on the attitude of European 198 pediatric oncologists and PPC doctors towards PPC confirmed these results (24). While 94.4% correctly disagreed that PPC leads to earlier death and 91.4% agreed that PPC could be integrated with disease-directed therapy, areas of lower alignment involved misconceptions about early PPC increasing family burden and uncertainty about the optimal timing for referrals. Thus, while 34% of physicians believed that PPC should be integrated at diagnosis, only 5% reported this occurring and 70% of physicians indicated that PPC typically starts when curative options are exhausted (24). The obstacles to timely integration included physician discomfort discussing PPC (82.3%), family resistance (72.2%), limited physician knowledge (71.2%), and uncertainty about prognosis (59.1%). Interestingly, respondents felt comfortable addressing physical (84.4%) and emotional needs (63.6%) but less comfortable with spiritual support (41.9%) and bereavement care (48.5%). Regional disparities were significant, with Southern and Eastern Europe reporting more issues related to access to home-based care, opioids, and PPC specialists (24).

In a survey on 158 pediatric oncology services from 27 European countries, the negative parental perception and late referrals were seen as the major barriers perceived by health care providers to implementation of PPC (25).

Misconceptions equating PPC with end-of-life care discourage families and healthcare providers from seeking or recommending these services (8). Different cultural attitudes regarding death and dying add complexity to these issues (9). Targeted education and awareness campaigns are essential to overcome these misconceptions, which delay referrals and worsen quality of life, especially for children with advanced or terminal cancer.Organizational barriers include a lack of trained professionals and challenges in integrating PPC with existing oncology care models (26). Insufficient funding and limited policy support reduce the availability of PPC services, particularly in underserved regions (26). This is also evident in Italy, where significant regional disparities exist in the availability of structured PPC services (27).

Another key organizational challenge is that children with complex or life-limiting conditions often face longer and more variable care trajectories than adults, involving frequent transitions between care settings (2). As a result, PPC teams must be structured flexibly to respond to these evolving needs across different environments, not limited to hospitals or home care alone.

Workforce development is a key component of improving PPC. The shortage of trained professionals underscores the need to integrate PPC into medical and nursing curricula and to establish ongoing education programs. Providing healthcare providers with specialized skills can greatly enhance the quality and accessibility of PPC services, as recommended by international standards (28). The shortage of healthcare professionals in pediatric palliative care is exacerbated by the high level of specialized training required and the profound emotional toll of working with critically ill children and their families, often leading staff to seek less demanding roles (29, 30).

## Models of development and organization in pediatric palliative care

Several models have been proposed for PPC organization. These may include floating clinics, disease-specific embedded teams, trigger-based supportive care clinics, telehealth clinics, and freestanding PPC clinics (31). Each model shows improvements in early PPC integration, symptom management, and patient-family communication. Telehealth may mitigate geographical barriers, while embedded and trigger-based teams promote proactive involvement of PPC (31).

In Italy, where significant disparities exist in PPC access, innovative and multifaceted models have been proposed to optimize resource allocation and improve care delivery. The choice of model depends on matching the children-family’s needs with available resources with an adaptable and resource-sensitive approach, provided the principles of timely referrals, equitable care, and tailored interventions are applied (32). Preliminary data collection is thus very important to inform the development and organization of these models (32).

Where regional networks for palliative care already exist, their expansion may ensure comprehensive territorial coverage and reduce disparities in underserved regions, particularly in southern and rural areas (8, 27). Some practical solutions models to improve access to PPC and integration with oncologic care are shown in **Table 1**.

**Table 1:** Some of the proposed models to integrate PPC into oncologic care for children with cancer (8, 18, 27, 32).

Model	Description	Advantages	Considerations/Limitations
Tiered Care	General pediatricians and oncologists manage routine PPC needs; specialized teams handle complex cases. Embedded PPC clinics within oncology units integrate palliative and cancer-directed care.	Efficient resource allocation, high-quality care, seamless coordination, concurrent care delivery.	Requires clear referral pathways and communication between generalists and specialists.
Home-Based Care	PPC delivered in the children’s home.	Aligns with family preferences, reduces institutional burdens, enhances patient and family satisfaction.	Requires robust home care infrastructure and support systems.
Telemedicine	Remote consultations and follow-ups.	Extends specialized care to underserved regions (especially rural areas), enhances continuity of care, bridges geographical gaps.	Requires reliable internet access and appropriate technology. Potential for limitations in physical examination.
Floating PPC Teams	Mobile teams providing consultative services across multiple hospitals and home-care settings.	Brings expertise to areas lacking dedicated PPC centers, valuable in low-resource settings.	Relies on investments in training, infrastructure, and effective coordination mechanisms. Potential challenges with continuity of care if teams are not consistently available.

Beyond institutional policies, public and professional engagement and education campaigns play a crucial role in promoting awareness about the existence of pediatric life-limiting diseases, including cancer, and about the role of PPC concerning disease-modifying therapies and end-of-life. These efforts aim to address misconceptions, such as equating palliative care solely with end-of-life care and emphasize its broader role in improving quality of life throughout the disease trajectory (9).

## The Italian landscape and local model

In Italy, the PPC network is structured on a regional and sub-regional basis, enabling each specialized center to serve a broad referral area, considering the estimated prevalence of children requiring specialized PPC, which is 18 minors per 100,000 population, with a median care duration of 44 months (10). This model is designed to address the need for highly specialized staff in PPC centers, including professionals such as pediatric anesthesiologists, pediatricians, nurses, and psychologists with specific expertise in pediatric care.

The recently published PalliPed 2022–2023 study offers a comprehensive assessment of the availability of these PPC centers across Italy, including for children with cancer (32). The study involved 18 centers from 14 regions and two autonomous provinces, with data collected through an online survey between January and May 2024.

Globally, children cared for by PPC teams rose from 1,209 (2019) to 2,734 (2023), meeting approximately 26% of the estimated 10,600 children requiring PPC annually. It is estimated that around 1800 of these are children with cancer (10). Three new regional centers were established since the inception of this initiative in 2021, bringing the total to 22 specialized PPC centers. Full territorial coverage was reported by 54% of referral centers, an improvement from 45% in 2021; however, only 23% of PPC centers provide 24/7 services, showing a decrease from 27%. This may be due to some of the participating centers recently starting their activity (32).

The most used model for PPC is home-based services (see **Table 1**), complemented by outpatient care and pediatric hospices, where available. PPC teams can be hospice-based or hospital-based. The workforce also saw notable growth, with nurses increasing by 38% and physicians by 62% since 2021. However, workforce shortages remain critical, with 77% of nurses lacking formal PPC training and most healthcare providers aged over 46. This is in line with the general shortage of health-care workers that Italy is facing. Significant gaps persist, particularly in southern Italy, where five regions still lack any specialized PPC services (32).

Among the new PPC centers developed during the period explored in the PalliPed 2022-2023 study, one is the PPC center in Brescia, Lombardia. The tertiary care university center of ASST Spedali Civili di Brescia includes a specialistic referral center for pediatrics which includes, among others, a pediatric oncology unit, a pediatric bone marrow transplant unit, and a pediatric ICU.

The PPC center began its activities in April 2023, supported by a team consisting of 2 PPC physicians, 3 nurses, and 1 psychologist. This center, while preserving its organizational and professional autonomy, is integrated into the palliative care network managed by ASST Spedali Civili, ensuring coordination with community services. The PPC center is hospital based and operates both within pediatric wards and in community settings, reflecting a mixed model of care (see **Table 1**). Although it does not have a dedicated hospice, the PPC center can admit children to the hospital pediatric clinic for treatment, caregiver training, or respite, based on the progression of the child’s condition. A key feature of this model is the “floating clinic” (see **Table 1**), which assesses children referred by specialists through shared screening tools, such as the “surprising question” and the “green lights” (33, 34). To provide homecare, the PPC center utilizes both home visits and telemedicine consultations. Telemedicine plays a crucial role, given the broad geographical area covered by the PPC center in Brescia and the need to involve multiple stakeholders in case discussions, such as social workers, local pediatricians, and school staff, alongside the child and family. In two years of activity, the center assisted a total of 84 children, including 10 children with cancer. Out of the total, 26 children passed away, including 5 with cancer. The maximum duration of care provided was 150 days, while for children with cancer, it reached 106 days (anonymous and aggregated data; unpublished). The higher mortality and shorter duration of care observed in children with cancer are consistent with findings from other centers and are likely attributed to the continuation of disease-specific treatments until advanced stages, as well as the misconception that pediatric palliative care is solely end-of-life care and mutually exclusive from active oncological treatments (20).

In our experience, many end-of-life situations, particularly for children with cancer, still occurred within the hospital due to families’ reluctance to face death at home and late referral. To address this, the PPC team collaborates with hospital pediatric teams to ensure a dignified end-of-life in a setting as comforting as possible for both the child and their family. Systematically supporting the hospital pediatric staff is important to meet this need, and to reduce the emotional burden that health-care professionals facing the death of a child. The presence of the PPC psychologist and, for some families, of the certified spiritual career adds quality to the holistic care provided to children dying from cancer. However, it is possible that earlier PPC would be associated with increased rates of death at home (35).

Our experience thus confirms that specialist PPC services for children with cancer can be effectively implemented even with a reduced staff, provided they are highly motivated and well-trained. Key organizational elements include screening for PPC eligibility by specialists, timely referrals, and comprehensive needs assessment using validated tools, such as the ACCAPED scale (36). Effective coordination with existing palliative and home care networks is essential (28). Hospital-based resources, such as designated beds or outpatient clinics, are important for enabling habilitation and respite admissions, as well as for reassessing needs and treating symptoms. Integration with hospital and community-based social services is crucial, especially in our context, where many pediatric palliative cases involve migrant families. These families often have larger households and face significant social and financial challenges, including housing instability.

## Conclusion

Pediatric palliative care is a vital component of comprehensive cancer care, proactively supporting children with cancer and their families. While Italy has made progress in establishing a legislative framework and developing PPC services, significant barriers remain in ensuring equitable access and consistent care throughout the trajectory of the disease. This results in fewer children with cancer referred to PPC than those with non-oncological conditions and at a later stage of disease. Addressing these issues requires a multifaceted approach, including workforce development, public awareness campaigns, and the implementation of innovative or flexible models of care delivery, particularly for low-resource settings. Continued advocacy, research, and financial investment are essential to further strengthen the Italian PPC system and ensure that all children with cancer receive the best possible care.
